# Supplementary figures and images for: Dynamic acceleration response of a rock slope with a horizontal weak interlayer in shaking table tests
Source: PLoS One. 2021 Apr 21;16(4):e0250418. doi: 10.1371/journal.pone.0250418 (PMC8059807; doi:10.1371/journal.pone.0250418)

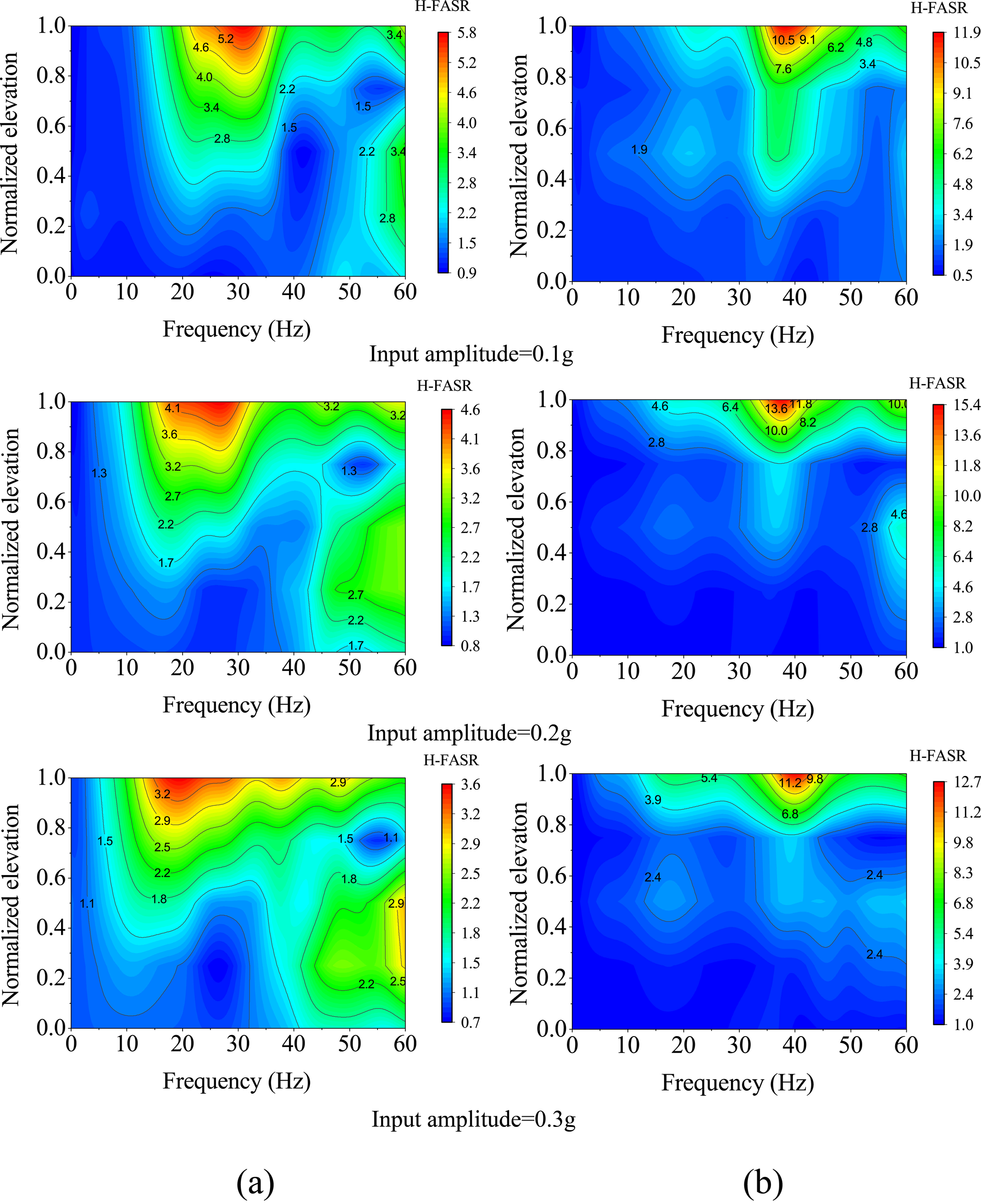

Supplement: S1 Fig — Fourier amplitude spectral ratios of horizontal accelerations (H-FASR) when input amplitude of horizontal shaking was lower than 0.3 g: (a) Slope A; (b) Slope B. (TIF) [file pone.0250418.s001.tif]

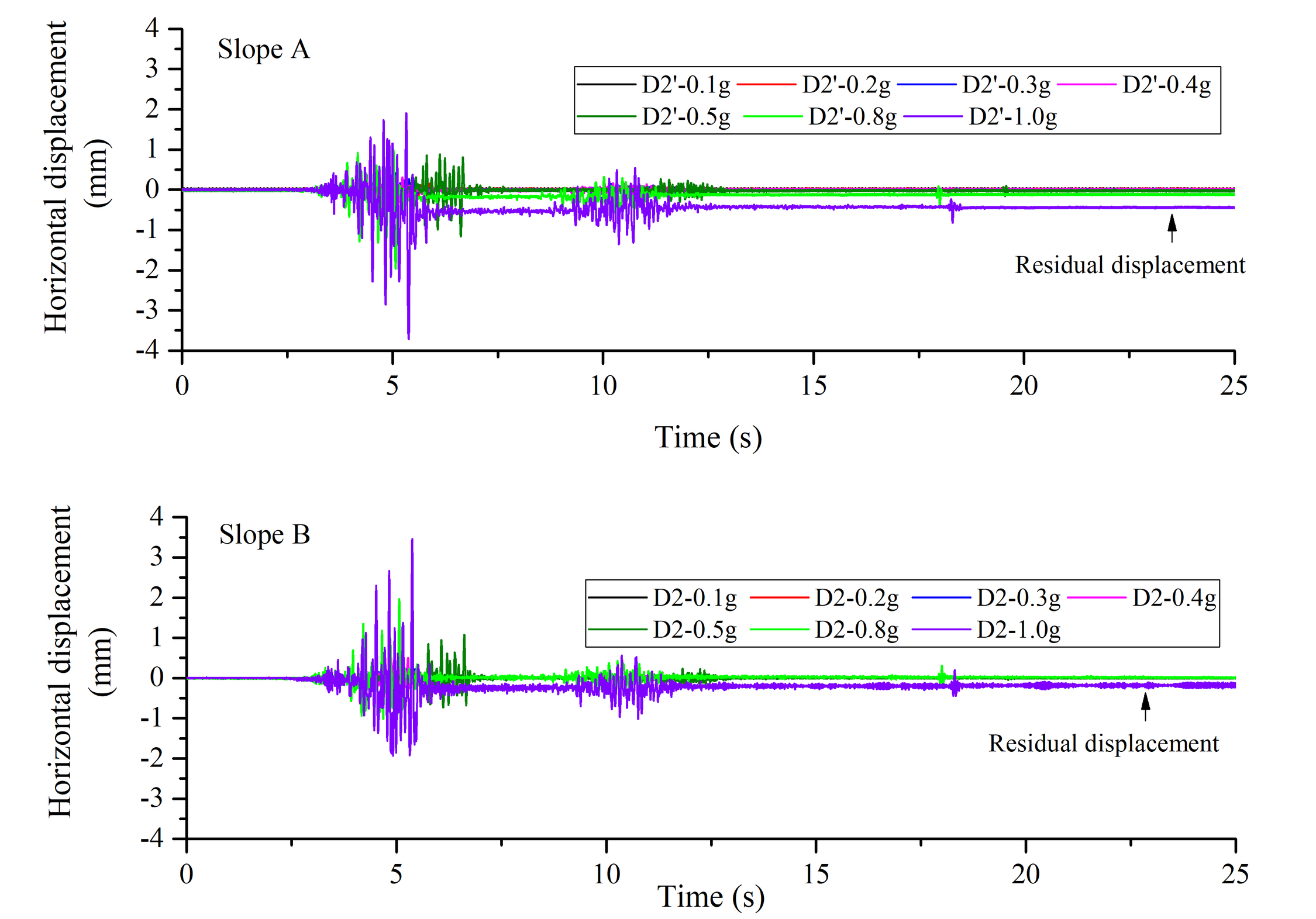

Supplement: S2 Fig — (TIF) [file pone.0250418.s002.tif]
