# Supplementary material for: Dynamic acceleration response of a rock slope with a horizontal weak interlayer in shaking table tests
Source: PLoS One. 2021 Apr 21;16(4):e0250418. doi: 10.1371/journal.pone.0250418 (PMC8059807; doi:10.1371/journal.pone.0250418)
Supplement: Attachment — Submitted filename: Response to Reviewers.docx [file pone.0250418.s003.docx]

**Response to Review Comments**

We have significantly revised our paper based on the constructive and helpful comments from the editors and reviewers. Our sincere thanks go to them for their suggestions and the opportunity for us to revise the manuscript. We have tried our best to address the review comments by making the following revisions. In the revised manuscript where the changes are tracked, all the revisions are highlighted in red.

**Response to Editors**

“1. *Please include the following items when submitting your revised manuscript:*

- *A rebuttal letter that responds to each point raised by the academic editor and reviewer(s). You should upload this letter as a separate file labeled* *'Response to Reviewers'.*
- *A marked-up copy of your manuscript that highlights changes made to the original version. You should upload this as a separate file labeled 'Revised Manuscript with Track Changes'.*
- *An unmarked version of your revised paper* *without tracked changes. You should upload this as a separate file labeled* *'Manuscript'.”*

**Response:** According to your instruction, three separate files have been uploaded: 'Response to Reviewers', 'Revised Manuscript with Track Changes', and 'Manuscript'.

*“2. If you would like to make changes to your financial disclosure, please include your updated statement in your cover letter.”*

**Response:** Thank you. No changes need to be made on the financial disclosure.

*“3. Guidelines for resubmitting your figure files are available below the reviewer comments at the end of this letter.”*

**Response:** Thank you. All figures were resubmitted using the files adjusted by the Preflight Analysis and Conversion Engine (PACE) digital diagnostic tool.

*“4. If applicable, we recommend that you deposit your* *laboratory protocols in* *protocols.io to enhance the reproducibility of your results.* *Protocols.io assigns your protocol its own identifier (DOI) so that it can be cited independently in the future. ”*

**Response:** Thanks for your suggestion. No laboratory protocols were deposited in protocols.io and the detailed descriptions of test program can be found in the section 'Test program' in our revised paper.

*“5. Please ensure that your manuscript meets PLOS ONE's style requirements, including those for file naming.”*

**Response:** The revised manuscript has been double-checked and submitted according to PLOS ONE’s submission guidelines and recently published papers in PLOS ONE.

*“5. Please include a caption for figure 3.”*

**Response:** Thank you for catching our mistakes. A caption for Figure 3 has been added in the revised manuscript. Please see **Page 9, Lines 222-224** in the revised manuscript without tracked changes.

*“6. Please include captions for your Supporting Information files at the end of your manuscript, and update any in-text citations to match accordingly.”*

**Response:** The supporting information files have been provided and cited in the revised paper according to the guidelines for Supporting Information in PLOS ONE.

**Response to Reviewer #1**

*“In general, the topic of the paper is certainly of interest to the researchers of slope stability under earthquake. However, there are some unclear statements and questions that need to be clarified. The comments are listed below.*

*1. The resolution of the graphs provided in the article is not clear. It is recommended that the author provide graphs with high resolution and good quality.”*

**Response:** Thanks for your suggestion. All figures were resubmitted using the files adjusted by the Preflight Analysis and Conversion Engine (PACE) digital diagnostic tool, with high resolution and good quality.

*“2. Do the material parameters of the physical model listed in table 3 conform to the simulation similarity law listed in table 2?”*

**Response:** Thanks for your comment. For the convenience of comparison, the ratios between prototype and model slopes for each parameter in Table 3 were calculated and shown in the following table. It can be seen that these ratios conformed to the corresponding scaling factors listed in Table 2, with an exception of cohesion, *c*. The cohesions of the rock and weak interlayer were much lower than the values required to satisfy the scaling factors of cohesion in Table 2. However, the dynamic behaviors of the model slopes, which are the focus of this present study, are not significantly influenced by the inconformity in cohesion, particularly under small deformations.

We have added the statements in the section "Preparation of model slopes" to address this issue, please see **Page 7, Lines 163-169** in the revised manuscript named as "Manuscript".

**Table 3. Physical and mechanical properties of prototype slopes and model slopes.**

| Lithology | | Density *ρ* (10^3^kg/m^3^) | Cohesion *c* (kPa) | Frictional angle *ϕ* (^o^) | Elastic modulus *E* (MPa) | Poisson’s ratio *μ* |
| --- | --- | --- | --- | --- | --- | --- |
| Rock | Prototype | 2.48 | 520 | 33.8 | 1900.0 | 0.31 |
|  | Model | 2.40 | 37.1 | 34.9 | 50.2 | 0.30 |
|  | Prototype/model | 1.0 | 14.0 | 1.0 | 37.8 | 1.0 |
|  | Scaling factor in Table 2 | 1.0 | 32.6 | 1.0 | 32.6 | 1.0 |
| Weak interlayer | Prototype | 1.60 | 30.0 | 35.0 | 41.0 | 0.35 |
|  | Model | 2.32 | 10.0 | 27.3 | 4.8 | 0.35 |
|  | Prototype/Model | 0.7 | 3.0 | 1.3 | 8.5 | 1.0 |
|  | Scaling factor in Table 2 | 0.67 | 10.67 | 1.0 | 10.67 | 1.0 |

*“3. A series of seismic loading tests are performed on the same model. How to ensure that the physical model will not affect the results of the next set of tests due to the deformation of the specimen, the generation of micro-cracks, and the interlayer sliding after the previous stage of the test?”*

**Response:** Thanks for your comment. You are right! It is widely known that loading history has a significant impact on the dynamic response of the model slopes. As a widely-adopted mitigation to reduce this impact, waves were applied to the model slopes with a gradually increasing amplitude [29-39]. The loading sequence in Table 4 allowed an insight into the evolution of the slope response from linear to nonlinear, assuming that the effect of minor deformation of model slope can be neglected under small-amplitude excitations. In addition, the cumulative damage caused by continuous loading could partially simulate pre-existing fractures/fissures in a prototype slope before a seismic event.

We have added the statements in the section "Input excitations" to address this issue, please see **Pages 8-9, Lines 205-212** in the revised paper named as "Manuscript".

*“4. In slope A, the thickness of the weak interlayer is 15 cm, E is 41 MPa (which is 1/10 of rock’s elastic modulus). Does the sliding between soft layer and rock or the deformation in the interlayer is observed during the first and second loading stages?”*

**Response:** Thanks for your comment. In the first stage, no sliding was observed between the weak interlayer and the surrounding rock, and no deformation was observed in the interlayer of both slopes. In the second loading stage, however, a small horizontal extrusion deformation occurred around the weak interlayer in both slopes, and the extrusion was more severe in Slope A (see Fig 15(C); S2 Fig) than in Slope B (see Fig 15(D); S2 Fig). The corresponding statements can be found in **Page 18, Lines 469-472** in the revised paper named as "Manuscript".

*“5. Figure 14 demonstrates the response features of the slope speculated according to the literature and this research. However, it is not clear how to the acceleration 0.3 g as a threshold value in Phase I.”*

**Response:** Thanks for your comment. We have to acknowledge that the cutoff values of input acceleration between two successive response phases of the model slope can’t be accurately determined just based on our current research. Nonetheless, we selected acceleration 0.3 g as the threshold value in Phase I, considering the approximatively linear response characteristics of both slopes as the input amplitude was lower than 0.3 g:

(1) Both the horizontal and vertical accelerations (e.g., PHA, R-PHA, PVA, R-PVA) at most monitoring points of the slopes generally increased as the input amplitude increased from 0.1 g to 0.3g (see Fig 7 and Fig 11).

(2) Minor change of resonant frequency occurred in both slopes as the input amplitude was increased from 0.1 g to 0.3g, suggesting a negligible structure deterioration of both slopes (see S1 Fig).

(3) No macro deformation occurred in any parts of the slopes.

The corresponding statements can be found in **Page 17, Lines 437-444** in the revised paper named as "Manuscript".

*“6. There is a horizontal truss in Figure 15 and the crack range shown in Figure 15(a)(b) does not seem to be the failure type developed by the tension crack on the top of the slope. Does the truss collide with the physical model of the slope during the seismic loading?”*

**Response:** Thanks for your comment. The truss didn’t collide with the model slopes during the seismic loading due to a sufficient horizontal distance between the truss and the slope surface (see Fig A1 below). The distance is larger than the maximum horizontal displacement out of slope (see S2 Fig).

**Fig. A1. Horizontal distance between truss and slope surface.**

**S2 Fig. Horizontal displacement time histories at monitoring points D2′ and D2 under different levels of horizontal shaking.**
