# Supplementary material for: Dynamic acceleration response of a rock slope with a horizontal weak interlayer in shaking table tests
Source: PLoS One. 2021 Apr 21;16(4):e0250418. doi: 10.1371/journal.pone.0250418 (PMC8059807; doi:10.1371/journal.pone.0250418)
Supplement: Attachment — Submitted filename: Comments.docx [file pone.0250418.s004.docx]

The dynamic response of a slope having a weak interlayer is an interesting but complex research topic in the slope earthquake engineering field. The research work in this paper can help understand the failure mechanism of geohazards with this type of slope structure under an earthquake. The model test program was designed rigorously and the results of model tests were analyzed in detail. The language is fluent and easy to understand, but there are still some aspects that need to be improved. My general comments are as follows:

(1) The shaking table test was conducted for a limited slope model geometry (regular single-side slope) and slope structure (a single horizontal interlayer). The authors are encouraged to carry out further research under more complex conditions (e.g., complex topography, slope structure, and seismic loading) so that the results obtained can be generalizable.

(2) As shown in Table 4, why are the maximum input amplitudes of vertical and horizontal accelerations different?

(3) Since the seismic input was from the Wolong station – the authors should explain the geological/geomorphological setting: is on the surface? on bedrock, what type? on flat/valley bottom area?

(4) Lastly, the present work focused on the acceleration responses of the slopes. It would have been nice to see some quantitative work on the failure mechanisms and failure modes of the slopes.
