# Supplementary material for: Dynamic acceleration response of a rock slope with a horizontal weak interlayer in shaking table tests
Source: PLoS One. 2021 Apr 21;16(4):e0250418. doi: 10.1371/journal.pone.0250418 (PMC8059807; doi:10.1371/journal.pone.0250418)
Supplement: Attachment — Submitted filename: Response to Reviewers.docx [file pone.0250418.s005.docx]

**Response to Review Comments**

We have significantly revised our paper based on the constructive and helpful comments from the editors and reviewers. Our sincere thanks go to them for their suggestions and the opportunity for us to revise the manuscript. We have tried our best to address them by making the following revisions. In the revised paper, all the revisions are highlighted in red.

**Response to Editors**

“1. *Please include the following items when submitting your revised manuscript:*

- *A rebuttal letter that responds to each point raised by the academic editor and reviewer(s). You should upload this letter as a separate file labeled* *'Response to Reviewers'.*
- *A marked-up copy of your manuscript that highlights changes made to the original version. You should upload this as a separate file labeled 'Revised Manuscript with Track Changes'.*
- *An unmarked version of your revised paper* *without tracked changes. You should upload this as a separate file labeled* *'Manuscript'.”*

**Response:** According to the guidelines for revisions, three separate files have been uploaded named as 'Response to Reviewers', 'Revised Manuscript with Track Changes', and 'Manuscript', respectively.

*“2. If you would like to make changes to your financial disclosure, please include your updated statement in your cover letter.”*

**Response:** Thank you. No changes need to be made on the financial disclosure.

*“3. Guidelines for resubmitting your figure files are available below the reviewer comments at the end of this letter.”*

**Response:** Thank you. All figures were submitted using the files adjusted by the Preflight Analysis and Conversion Engine (PACE) digital diagnostic tool.

*“4. If applicable, we recommend that you deposit your* *laboratory protocols in* *protocols.io to enhance the reproducibility of your results.* *Protocols.io assigns your protocol its own identifier (DOI) so that it can be cited independently in the future. ”*

**Response:** Thanks for your suggestion. No laboratory protocols were deposited in protocols.io and the detailed descriptions of test program can be found in Caption 'Test program' in our revised paper.

**Response to Reviewer**

*“The dynamic response of a slope having a weak interlayer is an interesting but complex research topic in the slope earthquake engineering field. The research work in this paper can help understand the failure mechanism of geohazards with this type of slope structure under an earthquake. The model test program was designed rigorously and the results of model tests were analyzed in detail. The language is fluent and easy to understand, but there are still some aspects that need to be improved. My general comments are as follows:*

*(1) The shaking table test was conducted for a limited slope model geometry (regular single-side slope) and slope structure (a single horizontal interlayer). The authors are encouraged to carry out further research under more complex conditions (e.g., complex topography, slope structure, and seismic loading) so that the results obtained can be generalizable.”*

**Response:** Thanks for your suggestion. You are right! The seismic slope responses are influenced by diverse factors such as topography, slope structure, and seismic loading. In the present study, we simplified the slope model geometry and the slope structure to highlight the effect of a weak interlayer on the complex seismic response and deformation/failure behavior of rock slopes. In the future work, an extensive numerical calculation will be carried out to study the seismic slope responses under more complex conditions using the numerical slope models calibrated by these shaking table test results, together with the parametric analysis. The reason for this simplification can be found in **Page 4, Lines 97-106** in the revised paper named as "Manuscript".

*“(2) As shown in Table 4, why are the maximum input amplitudes of vertical and horizontal accelerations different?”*

**Response:** Thanks for your comment. The target input amplitudes of vertical accelerations were designed to be the same with those of horizontal accelerations. However, the actual input amplitudes of vertical accelerations within the effective working frequency (< 80 Hz), which were collected by an accelerometer fixed on the shaking table, were lower than the target input amplitudes due to the capacity of the shaking table under high levels of vertical excitations. The target and actual input amplitudes of the vertical accelerations are shown in Table A1. The corresponding statement can be found in **Page 9, Lines 224-228** in the revised paper named as "Manuscript".

**Table A1. Targeted and actual input amplitudes of vertical accelerations in shaking table tests (revised from Table 4)**

| **loading step** | **Wave type** | **Target input amplitude (g)** | **Actual input amplitude (g)** | **Excitation direction** | **Duration (s)** | **Dominant frequency (Hz)** |
| --- | --- | --- | --- | --- | --- | --- |
| 1 | WL wave | 0.1 | 0.1 | Z | 27 | 32.4 |
| 3 | WL wave | 0.2 | 0.18 | Z | 27 | 32.4 |
| 5 | WL wave | 0.3 | 0.24 | Z | 27 | 32.4 |
| 7 | WL wave | 0.4 | 0.32 | Z | 27 | 32.4 |
| 9 | WL wave | 0.5 | 0.38 | Z | 27 | 32.4 |
| 11 | WL wave | 0.8 | 0.6 | Z | 27 | 32.4 |
| 13 | WL wave | 1.0 | 0.75 | Z | 27 | 32.4 |

*“(3) Since the seismic input was from the Wolong station – the authors should explain the geological/geomorphological setting: is on the surface? on bedrock, what type? on flat/valley bottom area?”*

**Response:** Thanks for your comment. We have added the following statement in the revised paper to describe the basic characteristics of the Wolong station:

**Page 9, Lines 217-224:** The input accelerations were scaled from the recorded accelerations at the Wolong seismic station (WL wave) during the 2008 Wenchuan earthquake, which was about 23 km southwest of the epicenter. The altitude on the station ground is 919 m above sea level whereas the altitude on the top of the mountain nearby is 3187 m. The overlying soils at the station are mainly composed of Quaternary alluvial and diluvial gravels and pebbles. The recorded accelerations had a dominant frequency of 2.4 Hz and 8.1 Hz in the horizontal and vertical direction, respectively, which were scaled to have dominant frequency of 9.6 Hz and 32.4 Hz, respectively (see Table 4).

*“(4) Lastly, the present work focused on the acceleration responses of the slopes. It would have been nice to see some quantitative work on the failure mechanisms and failure modes of the slopes.”*

**Response:** Thanks for your comment. We have to acknowledge that a completely quantitative analysis was not carried out with respect to the failure mechanisms and failure modes of the slopes, just based on the macroscopic observations in a shaking table test. The quantitative work on the seismic slope responses and failure modes and mechanisms will be carried out in the future using different research methods, including macro- and micro- numerical calculation, physical model testing, field monitoring, and theoretical analysis. We have added the corresponding statement in **Pages 18-19, Lines 487-491** in the revised paper named as "Manuscript".
